# Evaluation of oxidative stress markers in Rwanda during the SARS-CoV-2 pandemic: A cross-sectional study

**DOI:** 10.1371/journal.pgph.0002487

**Published:** 2023-10-25

**Authors:** Thierry Habyarimana, Alexis Nshimiyimana, William Niyonzima, Janvere Kankindi, Cedrick Izere, Chiara Spaggiari, Gabriele Costantino

**Affiliations:** 1 Department of Biomedical Laboratory Sciences, INES-Ruhengeri, Musanze, Rwanda; 2 Department of Food and Drugs, Università di Parma, Parma, Italy; PLOS: Public Library of Science, UNITED STATES

## Abstract

COVID-19 is mainly described as endothelial dysfunction, and due to the bidirectional link between oxidative stress and endothelial dysfunction, we initiated a program directed to the evaluation of the oxidative status of the population of Rwanda by measuring spectrophotometrically their plasma Reactive Oxygen Metabolites (d-ROMs) and Plasma Antioxidant Potential (PAT). The reference population was chosen to reflect the absence of actual or past SARS-CoV-2 infections as well as other clinically established infective status and reference intervals for d-ROM and PAT were identified. The average d-ROM was 378.6 UCARR with a standard deviation of 105.2, a value significantly higher than that reported for Caucasian or East Asian population (260–300 UCARR). The average PAT value was 2853.6, with a standard deviation of 635.7 UCOR, at the upper limit according to the averaged values for healthy Caucasian populations. The results of this study, the first so far reported on a sub-Saharan population, can effectively be used as a baseline value for clinical management of inflammatory conditions, for the stratification of at-risk individuals and to inform recommendations for effective use of public health resources.

## Introduction

The emergence of SARS-CoV-2 at the beginning of 2020 and the subsequent COVID-19 pandemics urged countries and national health systems to identify quick, cheap, and effective tools not only for diagnosing the infections but also to stratify those subjects who can be at higher risk of a negative outcome. This holds particularly true for low/middle-income countries, where it is fundamental to reduce unnecessary burdens on the public health systems [1]. In 2021, the Republic of Rwanda, in East Africa, funded a research program aimed at stratifying the population according to the risk of a negative outcome after SARS-CoV-2 infection thus helping to allocate the medical resources where needed and to decrease the use of non-pharmacological measures which could negatively impact the economy and the societal activities.

Although generally described as a severe and acute respiratory syndrome, COVID-19, the clinical manifestation of the SARS-CoV-2 infection, often involves a variety of other organs and tissues, including the micro- and macro-vascular system, heart, liver, kidney, spleen, testes, and central nervous system (CNS) [2, 3]. A possible and attractive unifying explanation for all these phenomena is the presence of endothelial dysfunction [4]. Endothelial dysfunction is a general term that covers the reduced or impaired ability of endothelial cells to produce nitric oxide (NO) and the subsequent unbalanced production of Reactive Oxygen Species (ROS) and endothelium-derived contracting factors. Thus, SARS-CoV-2 can attack endothelial cells which abundantly express Angiotensin-converting enzyme 2 (ACE2) receptors, causing vasoconstriction, inflammation, cytokine storm, coagulopathy, and production of mitochondrial ROS. In turn, individuals who already have endothelial dysfunctions because of underlying conditions (e.g., obesity, diabetes, coronary diseases, and metabolic syndrome) may catastrophically exacerbate the condition after SARS-CoV-2 infection [5]. Since endothelial dysfunction is bidirectionally linked to oxidative stress and ROS production [6], we reasoned that the evaluation of the oxidative status can be a cheap, easy, and reliable way to stratify people at risk of exacerbating endothelial dysfunction and thus developing severe diseases. We thus initiated a program, whose first results are herein reported, directed to evaluate the oxidative status of the population of the Northern Province of Rwanda, Musanze District, by using the dROM/PAT approach, a cheap, noninvasive, and quick method to assess the oxidative status (dROM) and the antioxidant potential (PAT) of plasma collected with a finger prick [7–9].

The d-ROMs (derivative-Reactive Oxygen Metabolites) test measures spectrophotometrically the total amount of organic peroxides in a blood sample [7, 10–15]. An oxidative stress condition can be compensated by an efficient antioxidant system and for this reason, the evaluation of d-ROMs must be accompanied by the valuation of the antioxidant potential of the plasma (PAT) [9]. Individuals having a high antioxidant potential (PAT) can better buffer high d-ROMs. PAT of a blood sample can conveniently be measured spectophotometrically by evaluating the blood’s ability to quench a solution of Fe3+. For Caucasian populations, PAT values around 2800–2200 UCOR (1 UCOR corresponding to 1.4 μmol/ml of ascorbic acid) are normal, while values lower than 1800 are indicative of insufficient antioxidant competence.

Despite some known limitations [16], the d-ROMs test is increasingly used and recognized as a useful predictor of increased risk of cardiovascular events and recently it has been successfully used to stratify severe outcomes of SARS-CoV-2 in Slovenia as well as in Macedonia, Romania, Italy and Greece to assess the oxidative stress parameters among hospitalized COVID-19 patients [17–21]

An important limitation of the d-ROMs (PAT) test is the lack of universally accepted reference intervals for the UCARR (UCOR) values. In particular, previous studies indicate an interval between 260–310 UCARR and 2200–2800 UCOR as normal ranges for d-ROMs and PAT respectively, in healthy Caucasian populations [7]. Nevertheless, since the reference intervals have to be used as ‘healthy intervals’ in the context of Rwanda and Central East Africa more in general, they should be based on a reference population which can be used as control for further screening campaigns and which takes into account the specificity concerning age distribution, gender, ethnicity.

On the basis of these consideration and also given the known differences in some clinical-chemical read-outs between sub-Saharan Africans and Caucasian counterparts [22], we proceeded to assess the reference intervals in oxidative status (d-ROMs) and antioxidant potential (PAT) in the population of the Musanze district through a cross-sectional observation study, as the basis for future stratification of individuals for the risk

## Materials and methods

### Geographical and demographic context

The study has been carried out in the district of Musanze, Northern Province of Rwanda. Rwanda is a land-locked country, with approximately 13 million inhabitants. The region is mainly hilly and rich in water. The average age is 20.4 years (2020), and the life expectancy is 68.6 years.

### Ethical considerations

The study was conducted according to the guidelines of the Declaration of Helsinki, and approved by Rwanda National Ethics Committee (No.90/RNEC/2021 of 23rd March 2021). In addition, the authorization to conduct this study has been provide by the Northern Province (No. 308/07.04/AAG/2021). Informed consent was obtained from all subjects involved in the study.

### Inclusivity in global research

Additional information regarding the ethical, cultural, and scientific considerations specific to inclusivity in global research is included in the Supporting Information (S1 Checklist).

### Sample collection

Samples were collected during three individual sessions held at INES-Ruhengeri—Institute of Applied Sciences and at the city markets between March and June 2021. A total of 1305 volunteers were screened. Visitors and workers in three spots were asked to voluntarily participate to the screening. Only people who were not positive for and did not report previous SARS-CoV-2 infections were included. A rapid antigenic SARS-Cov2 test was performed before sample collection. After a demographic questionnaire and a basic anamnesis, a drop (around100 μL) of capillary blood was collected by pricking the finger and storing it in heparinized microcuvette. The microcuvette was immediately centrifuged (2500 rpm 141 for 1 min) in order to isolate the plasma on which the test was then performed.

### Measure of the d-ROMs

Briefly, the serum was added to an acidic buffered solution (R1 reagent) in a cuvette; then, a colorless oxidizable chromogenic mixture (R2 reagent) was added. After mixing well and setting the cuvette in the analyzer, absorbance change at 505 nm was calculated. The change in absorbance is then transformed in UCARR by applying a correction factor as specified by the manufacturer. The test was carried out by using the dedicated FRAS 5 system (H&D srl Parma, Italy [9].

### Measure of the PAT

40 μL of R2 reagent (iron solution) was added to the cuvette containing R1 reagent (thio- cyanate derivate pre-dosed solution), followed by 10 μl of the serum sample. The reading was taken at 505 nm following a 1-minute incubation period at 37°C using the dedicated FRAS 5 system (H&D srl, Parma, Italy [8]. The change in absorbance is then transformed in UCOR units by applying a correction factor as specified by the manufacturer.

### Statistical analysis

Normality of the distributions was assessed by the skewness and kurtosis test. The upper and lower limit of the 95% reference intervals were determined by adding and subtracting, respectively, 1.96 x S.D to the mean of the distribution. 90% confidence intervals (C.I.) for the upper and lower reference limits were calculated from the normal distributions by using: C.I. = limit+/- 1.64 x SQRT(variance(1/n+2/n-1)). For comparison of means an independent t-test was used and statistically significant differences are given at p<0.05.

## Results

### 1. Demographic analysis of the sampled populations

A total of 1305 blood samples were collected in two different sites during three sessions within the district of Musanze. Of these, 554 came from female and 751 from male individuals.

**Fig 1:**
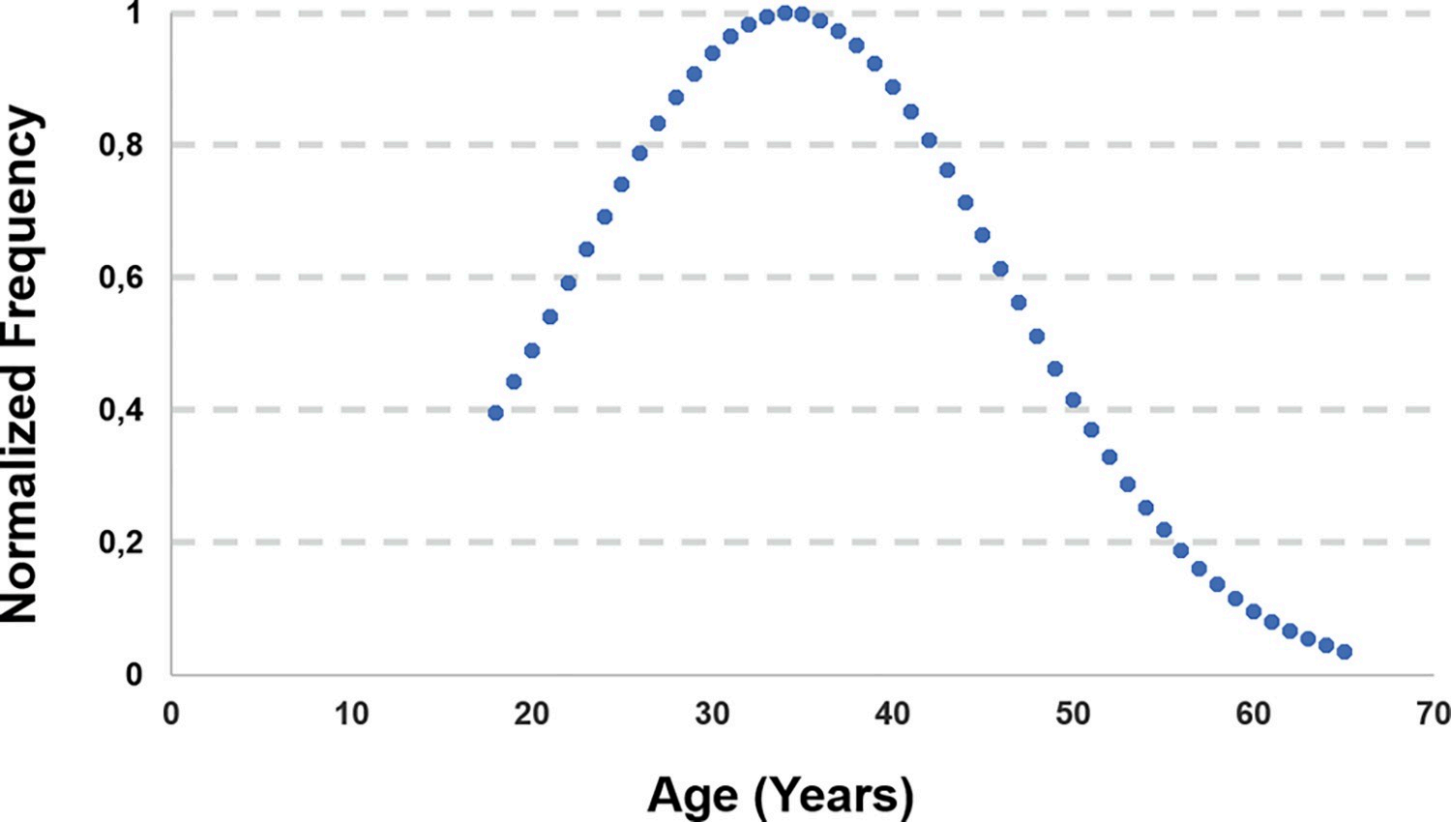
Age structure of the studied population.

The age of the whole dataset is normally distributed with an average age of 34.2 years old and a standard deviation of 11.9 (Fig 1). The distribution is truncated between 18 and 65 years old, as this was the chosen limit for recruiting volunteers. It should be observed that given the age structure of Rwanda, the sampled population is representative of about 56% of the population, being more than 40% of the population younger than 16 years old, and only the 3% older than 65. As a result, the average age of the country is estimated to be 20.4 years old.

### 2. d-ROMs test

**Fig 2:**
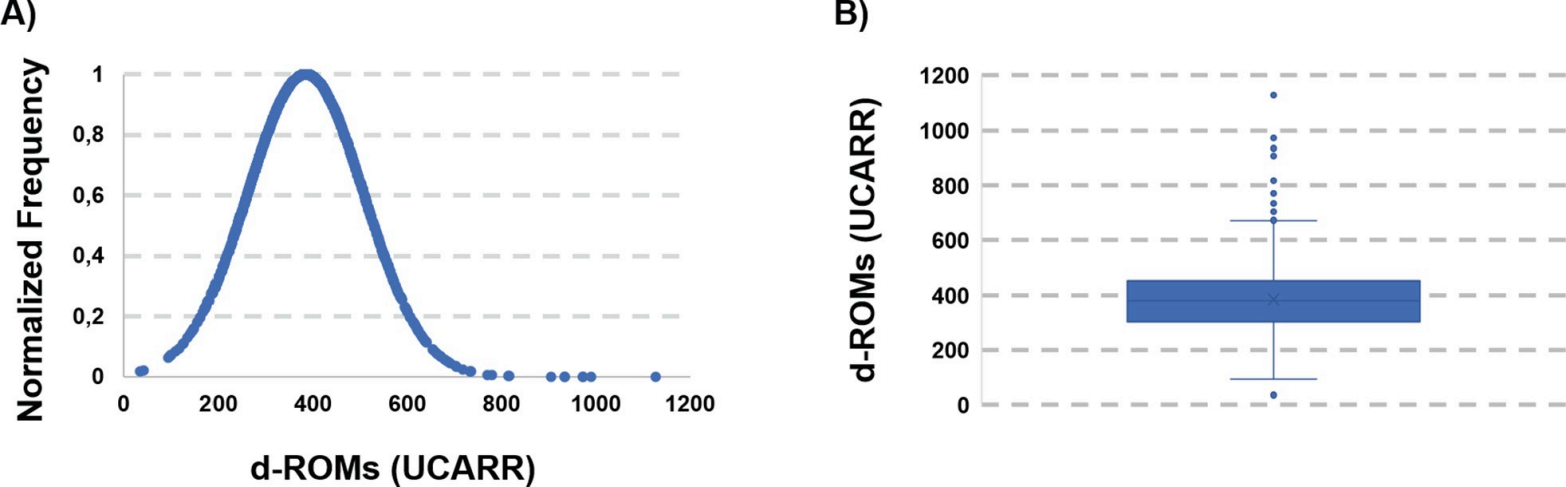
**A) Distribution of d-ROMs in the studied population.** The distribution is asymmetric with a long tail toward high d-ROMs values. **B) Box Plot of the d-ROMs distribution.** The Interquartile Range (IQR) is narrow. Values outside 1.5*IQR+third quartile and first quartile– 1.5*IQR were considered outliers and removed from further analysis.

Evaluation of the oxidative status of the whole set of the collected blood samples afforded an asymmetric Gaussian-type distribution (Kurtosi = 9.14, Asymmetry = 1.51, Fig 2A).

The average d-ROM was 384.4, with a standard deviation of 123.1. The value is significantly higher than expected on the basis of the available literature, as this value would correspond to a medium-high oxidative stress status according to the average values for a healthy -Caucasian- population. Before drawing conclusions, however, we investigated the reason for the asymmetric Gaussian-type distribution. Closer inspection revealed that the interval of the registered d-ROMs values is very broad (1542 UCARR units) and that the minimum and maximum values are very unusual, being 35 and 1577 UCARR, respectively. To detect and exclude possible outliers (possibly due to technical errors of the operators) we constructed a Box Plot (Fig 2B).

**Fig 3:**
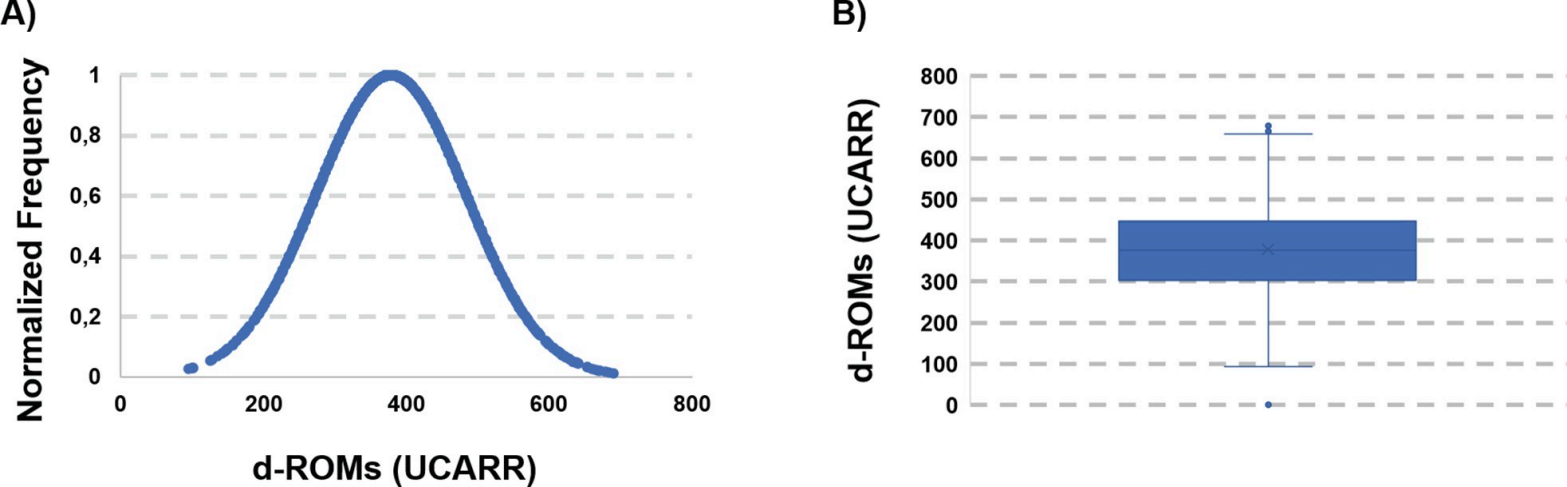
A) d-ROMs distribution after removal of the outliers. B) Box-plot of the pruned dataset.

According to the calculated Interquartile Range (IQR), we decided to consider as outliers those measures which are below the first quartile minus 1.5*IQR and above the third quartile plus 1.5*IQR. This resulted in the elimination of the samples with d-ROMs > 670 UCARR and d-ROMs< 83 UCARR. The pruned data, containing now 1260 observations (532 female and 728 male), was then reanalyzed. Elimination of the outliers resulted in a slight decrease of the average d-ROMs (378.6 UCARR, standard deviation 105.2) which is nevertheless still remarkably high. The distribution curve (Fig 3A) is now symmetric (asymmetry = 0.21, kurtosis = -0.169) as it is the box-plot (Fig 3B).

### 3. PAT test

**Fig 4:**
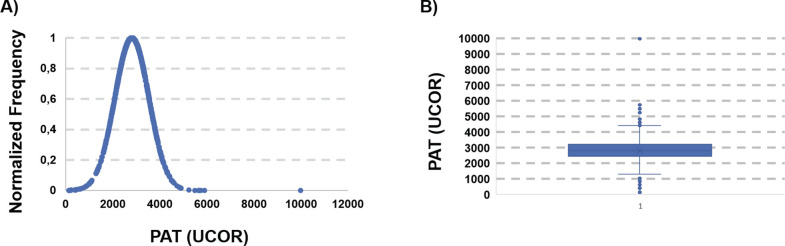
A) distribution of the PAT values of the whole dataset. B) Box-Plot. Values outside 1.5*IQR+third quartile and first quartile– 1.5*IQR were considered outliers and removed from further analysis.

The same procedure was repeated for the analysis of the antioxidant potential (PAT) of the collected blood samples. The average PAT value for the 1305 suitable samples was 2815.7 UCARR, which is comparable with published data for healthy Caucasian populations. Nevertheless, also in this case the analysis of the distribution curve (Fig 4A) and of the box-plot (Fig 4B) revealed a very broad interval (9856 UCOR), with extreme points (132 and 9988, respectively).

**Fig 5:**
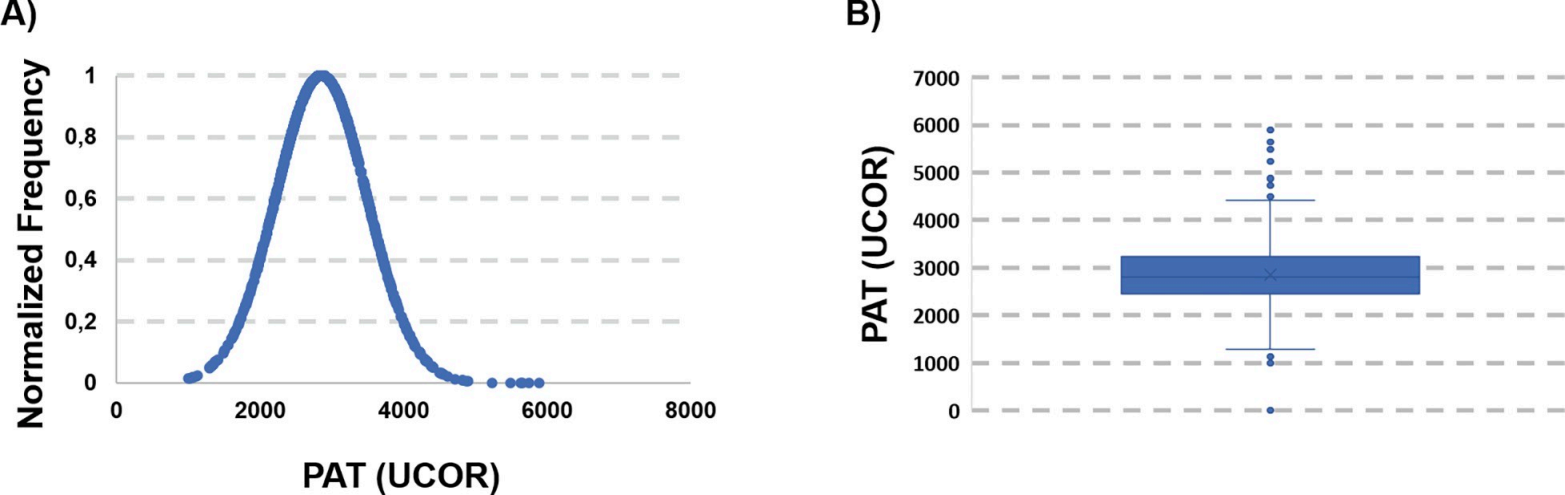
PAT distribution after removal of the outliers.

Also, in this case, the box-plot and the evaluation of the IQR suggested to eliminate a number of outliers (PAT < 950 and PAT >4500). The analysis was then repeated, and the pruned dataset contained 1261 samples, normally distributed around an average value of 2853.6, with a standard deviation of 635.7 (Fig 5). This average value is at the upper limit of what is considered normal according to the published data for healthy Caucasian populations.

### 4. Gender differences

It is reported that females in their fertile age have higher d-ROMs values than males [23, 24] although females seem to be less prone to oxidative stress than males, possibly because of the protective role of estrogens [25]. We have therefore split the pruned datasets (for d-ROMs and PAT, respectively) into a female and male dataset to check whether such differences are present.

**Fig 6:**
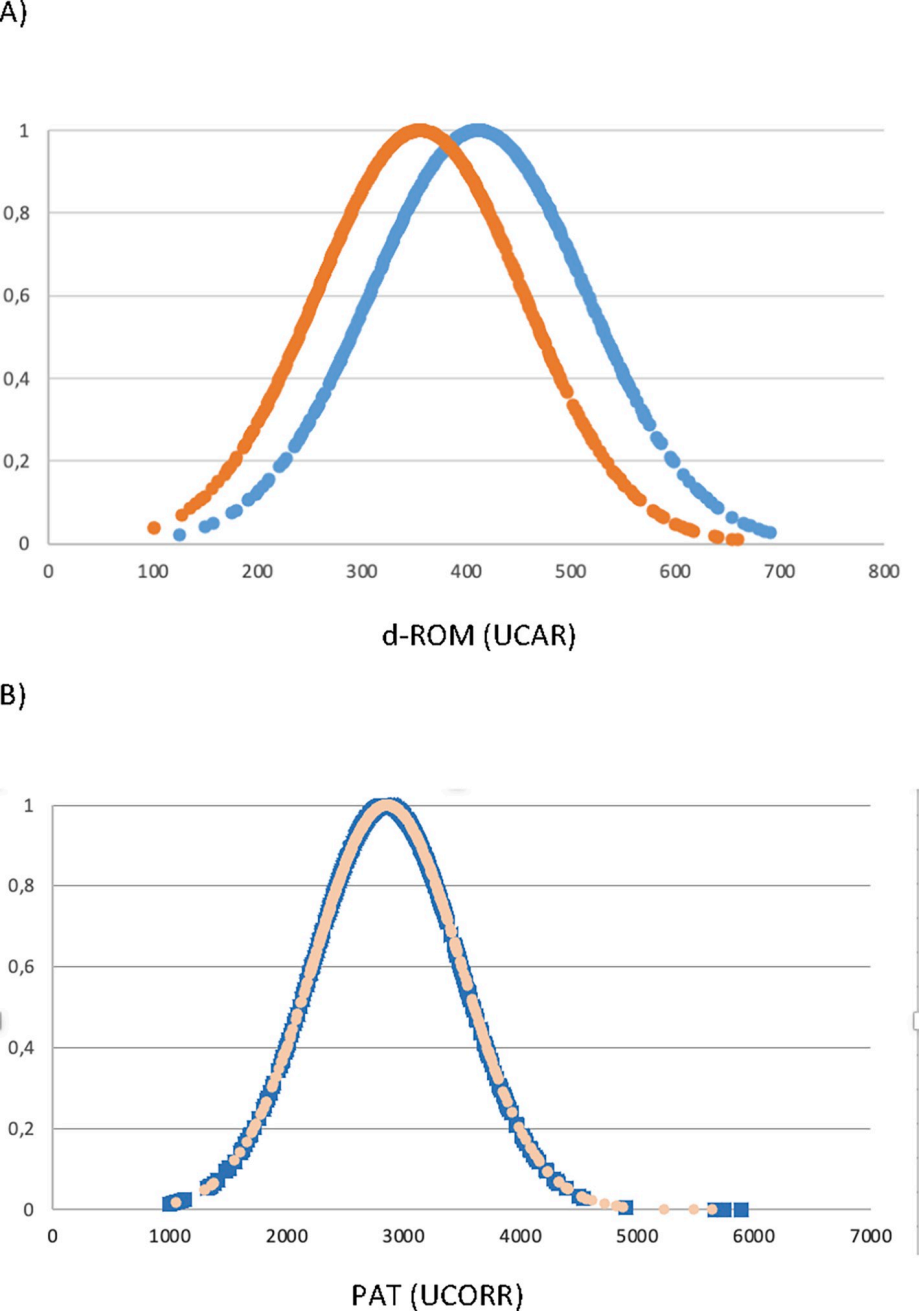
A) d-ROMs and B) PATs for the male (blue) and the female (pink) subgroups.

The 532 female individuals (average age 31 years old) had an average d-ROMs of 411.3 (standard deviation, 103.7) UCARR. The PAT tests conducted in the same dataset gave an average value of 2857.3 (standard deviation, 634.5) UCOR. The values obtained for the 728 male individuals (average age 32 years old) are 355 (standard deviation, 99.8) UCARR for the d-ROMs and 2850.5 (standard deviation 637.4) UCOR for the PATs. Results are summarized in Fig 6.

Table 1 reports the obtained reference intervals for the reference population, along with the confidence intervals for the upper and lower limits of the reference intervals.

**Table 1:** Reference intervals for d-ROMs and PATs, for the total reference population, and for the male/female subsets.

		Mean	Median	Standard Deviation	Lower Limit (*C*.*I*. *90%)*	Upper Limit (*C*.*I*. *90%*)
**Total Reference Population (n = 1260)**						
	dROMs (UCARR)	**378.6**	377	105.2	**172.4**(*164*.*0–180*.*8*)	**584.8** (*575*.*4–593*.*2*)
	PAT (UCOR)	**2853.6**	2796	635.7	**1572.3** (*1556*.*3–1588*.*4*)	**4134.0** (*4118*.*9–4150*.*9*)
**Male (n = 727)**						
	dROMs (UCARR)	**355.4** *	353*	99.1	161* (*150*.*6–171*.*45*)	549.8* (*539*,*35–560*,*25*)
	PAT (UCOR)	**2852.6**	2810	629.9	**1630.6** (*1564*.*2–1697*.*0*)	**4074.5** (*4008*.*1–4140*,*9*)
**Female (n = 533)**						
	dROMs (UCARR)	**411.3** *	409.5*	103.7	208.2* (*195*.*4–221*.*0*)	614.6* (*601*.*8–627*.*4*)
	PAT (UCOR)	**2857.3**	2762	634.5	**1613.7** (*1535*.*6–1691*.*9*)	**4100.9** (*4022*,*8–4178*.*2*)

*Statistically different at p<005

The most interested result here is the clear difference of the d-ROMs average values between the male and female subgroups. Indeed, the two groups belong to different statistical distribution and the average d-ROM is significantly different at p<0.05. Thus, the female population has a significantly higher oxidative status than its male counterpart.

Even more interesting is that the two populations have an age structure not statistically different at p<0.05 and, furthermore, that the antioxidant potential of their blood as exemplified by the PAT values, is statically identical at p<0.05. Thus, the female population is significantly imbalanced towards an oxidative stressed status with respect to the male population.

### 5. Post-hoc analysis

During the sample collections, volunteers were interviewed for basic health status. In particular, it was registered whether the volunteer is a smoker, is a frequent alcohol consumer, has or has not received an anti-SARS-CoV-2 vaccine shot. In addition, female participants were asked whether they assume oral contraceptives. Finally, the volunteers were asked to declare the presence of non-transmissible conditions, not requiring hospitalization, such as hypertension, diabetes, dyspepsia, asthma, and so on.

We thus investigated whether there is a relation or trend between the d-ROMs and PAT values and these basic health status indicators.

**Fig 7:**
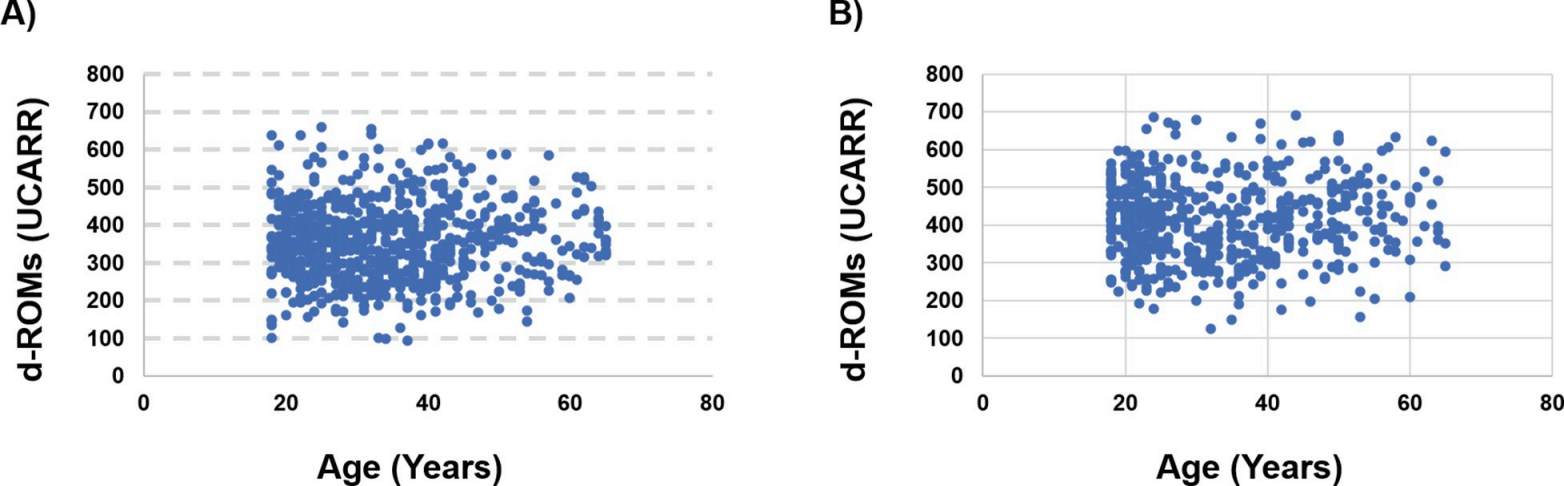
Dependence of d-ROMs from age in the two subgroups. A) Male and B) Female.

First, we noticed that in both male and female subgroups, there is no relationship between d-ROMs and age, or PAT and age (Fig 7). This agrees with previous reports that the oxidative status is almost constant during life in the absence of disease [26, 27].

Then we looked for trends with basic health status indicators. Reference individuals were in fact randomly recruited from the reference population which included citizens (18–65 years old) with no present of previous SARS-CoV-2 infections, nor with other clinically relevant infectious diseases. 135 individuals self-declared non transmissible conditions (referred to as in the questionnaires like ‘stomach diseases’, ‘diabetes’, ‘liver’, ‘asthma’). This subset had significantly higher d-ROMs values (average 403.6, standard deviation 103.46 UCARR) with respect to the non-reporting subset 377.1 (n = 1127, standard deviation 105.24 p<0.01) while having a non-significant difference in their PAT values (average 2843,8 ±723.4 (n = 135) Vs 2853.17±624.3 (n = 1177, p<0.5), a clear indication of an oxidative stress status. While the self-reported status and the lack of clinical specifications hamper a robust analysis of this data, this is in line with previous reports of d-ROMs (and even more the ratio d-ROM/PAT) as a proxy marker for non-transmissible conditions such as cardiovascular or metabolic diseases [28, 29].

Then we investigated the male dataset for alcohol/no alcohol, and SARS-CoV-2 vaccination status. The number of smoker volunteers was so small that the statistical analysis was not carried out. Interestingly, we observed that people who regularly consume alcohol have higher d-ROMs than people who are not used to drinking. The average d-ROM in the first group is 360.9 versus 347.1 in the second one (p<0.05).

We could only identify a trend when the SARS-CoV-2 vaccination status was examined. Vaccinated people have higher d-ROMs (362.9 UCARR) than non-vaccinated ones (353.5), but the difference was not statistically significant. Finally, when the female dataset was studied, volunteers who declared to use oral contraception had much lower d-ROMs (399.0 UCARR) than individuals who were not under oral contraception (415.4 UCARR). The difference is significant at p<0.08. This is in sharp contrast with previous reports which associated estro-progestinic treatment with an increase in oxidative status [23, 24].

## Discussion

This is, to the best of our knowledge, the first systematic study of the oxidative status of a large sample of a population in equatorial Africa. While differences between ethnicities have been reported in the past among Caucasians, Afro-Americans, Hispano-Americans and East-Asians [25] the present results give a more systematic analysis and provide an operational basis for the use of the d-ROMs and PAT tests for screening/follow-up purposes in sub-Saharan Africa. This is particularly relevant in the context of public health preservation and of optimal allocation of public health systems resources. First of all, it should be observed that the FRAS technology and the measure of d-ROMs and PAT as indicators of the oxidative status and of the antioxidant potential of the plasma, respectively is only one of the possible approaches for the measure of the oxidative stress. Some of the limitations of the test have been discussed by others [16], and are mainly related to the proxy nature of the d-ROM, which is an undirect measure of the reactive oxygen species, and to the lack of a universally accepted interval of normality for the read-out, being this latter issue partially addressed in this paper. Nevertheless, it should be also mentioned that the FRAS technology and the measure of the d-ROMs and PATs have several advantages. It has a low intra-assay variability which ensures reproducibility, and it is a cheap, not invasive, and quick measure which makes it particularly attractive for population screening in geographical and social contexts of low resources.

The first clear indication we obtained is that the oxidative status, as measured by the d-ROMs parameter, is significantly higher than that observed in analogous population-wide studies in Europe or in East Asia. The average d-ROMs, corrected after removal of some outliers, is 378.6 UCARR, (standard deviation 105.2). The interval of normality for European Caucasian individuals, with no underlying medical conditions, is 250–300 UCARR [7].

The reasons for this large difference can be attributed to several factors, including dietetic habits and climate, but the potentially most relevant explanation is the widespread exposition of the population to parasitic infections [30–32]. Production of ROS is the organism’s first line of defense against these communicable diseases and the higher level of peroxidation products found in the sub-Saharan population relative to the Caucasian counterpart is likely to reflect this concept. When the antioxidant potential, as measured by the PAT test, is evaluated, it is centered around a value of 2853.6 UCOR, with a standard deviation of 635.7. This is still within the range of normality accepted for Caucasian populations but at the upper level. This can be tentatively interpreted as an adaptation to a more oxidant environment: people produce larger amounts of ROS but have a higher ability to quench them.

Another clear indication that we extracted from this study, is that the female subgroup has a significant higher d-ROMs status than the male counterpart. This is not counterbalanced by an analogously higher antioxidant potential (PAT), which is indeed statistically identical in the two gender subgroups. This suggests that the male subgroup should be taken as the ‘control group’ if reference ranges for normality will be requested for future studies. The difference between male and female groups, furthermore, offers a first important public health policy indication: the female population is more susceptible to oxidative stress, which may be reflected for example in a high occurrence of post-menopausal cardiovascular events, and should be compensated by appropriate change in diet, including the use of antioxidant supplements.

Finally, we note the high standard deviations associated with the average values, which are reflected in quite broad reference intervals despite the high numerosity of the sampled population. The reason for this is not immediately apparent and certainly calls for further studies in order to assess the biological nature of the inter-individual variability. It should be however noted that d-ROMs and PATs are biochemical and not clinical markers, and their use in population screening is finalized at the early identification of extreme situation which can be suggestive of infective or chronic conditions. At the individual level, on the other side, is the variation from the own’s initial value which is indicative of progression or regression from a pathological condition more than the actual absolute value.

## Conclusion

This study has some limitations. Its observational cross-sectional character, coupled with the self-reported health status does not allow to exclude the presence of active infections in some individuals which may increase the level of ROS. The second limitation is inherent to the nature of the d-ROMs measurement, which only provides a proxy of the oxidative status, being a measure of the total peroxy-derivatives present in the plasma and not a direct measure of ROS. The large amount of the sampled population, and the robust statistics we obtained, make us confident that the observed measurements can effectively be taken as a baseline value for further screening purposes, either in the context of the COVID-19 pandemic or in other conditions, such as malnutrition or other noncommunicable chronic diseases, with the aim of productive population stratification and correct use of available medical resources.

## Supporting information

S1 Checklist Inclusivity in global research. (DOCX)

S2 Checklist STROBE statement. (PDF)

S1 Data Dataset. (XLSX)

S1 File Approval letter. (PDF)

S2 File Authorization. (PDF)
