# Supplementary material for: Evaluation of oxidative stress markers in Rwanda during the SARS-CoV-2 pandemic: A cross-sectional study
Source: PLOS Glob Public Health. 2023 Oct 25;3(10):e0002487. doi: 10.1371/journal.pgph.0002487 (PMC10599508; doi:10.1371/journal.pgph.0002487)
Supplement: S2 Checklist — (PDF) [file pgph.0002487.s002.pdf]

Manuscript PGPH-D-23-00887

STROBE Statement—checklist of items that should be included in reports of observational studies

	Item No.	Recommendation	Page No.	Relevant text from manuscript
**Title and abstract**	1	(*a*) Indicate the study's design with a commonly used term in the title or the abstract	1	Evaluation of Oxidative Stress Markers in Rwanda during the SARS-Cov2 Pandemic: A Cross-sectional Study
		(*b*) Provide in the abstract an informative and balanced summary of what was done and what was found	2	we initiated a program directed to the evaluation of the oxidative status of the population of Rwanda by measuring spectrophotometrically their plasma Reactive Oxygen Metabolites (d-ROMs) and Plasma Antioxidant Potential (PAT).<br>The average d-ROM was 378.6 UCARR with a standard deviation of 105.2. The average PAT value was 2853.6, with a standard deviation of 635.7 UCOR.
**Introduction**				
Background/rationale	2	Explain the scientific background and rationale for the investigation being reported	3-4	An oxidative stress condition can be compensated by an efficient antioxidant system and for this reason, the evaluation of

				d-ROMs must be accompanied by the valuation of the antioxidant potential of the plasma (PAT). Individuals having a high PAT can better buffer high d-ROMs. since the reference intervals have to be used as ‘healthy intervals’ in the context of Rwanda and Central East Africa more in general, they should be based on a reference population which can be used as control for further screening campaigns and which takes into account the specificity concerning age distribution, gender, ethnicity.
Objectives	3	State specific objectives, including any prespecified hypotheses	4	Given the known differences in some clinical-chemical read-outs between sub-Saharan Africans and Caucasian counterparts, we proceeded to assess the reference intervals in oxidative status (d-ROMs) and antioxidant potential (PAT) in the population of the Musanze district, as the basis for future stratification of individuals for the risk of developing a severe form of infectious diseases such as COVID-19.

**Methods**				
Study design	4	Present key elements of study design early in the paper	5-6	Geographical and demographic<br>Ethical considerations<br>Sample collection<br>Measurement of the d-ROMs<br>Measurement of the PAT<br>Statistical Analysis
Setting	5	Describe the setting, locations, and relevant dates, including periods of recruitment, exposure, follow-up, and data collection	5	Geographical and demographic<br>Ethical considerations<br>Sample collection
Participants	6	(*a*) *Cohort study*—Give the eligibility criteria, and the sources and methods of selection of participants. Describe methods of follow-up<br>*Case-control study*—Give the eligibility criteria, and the sources and methods of case ascertainment and control selection. Give the rationale for the choice of cases and controls<br>*Cross-sectional study*—Give the eligibility criteria, and the sources and methods of selection of participants	5	Sample collection
		(*b*) *Cohort study*—For matched studies, give matching criteria and number of exposed and unexposed<br>*Case-control study*—For matched studies, give matching criteria and the number of controls per case		
Variables	7	Clearly define all outcomes, exposures, predictors, potential confounders, and effect modifiers. Give diagnostic criteria, if applicable	5-6	Sample collection<br>Measurement of the d-ROMs<br>Measurement of the PAT
Data sources/ measurement	8*	For each variable of interest, give sources of data and details of methods of assessment (measurement). Describe comparability of assessment methods if there is more than one group	5-6	Sample collection<br>Measurement of the d-ROMs<br>Measurement of the PAT
Bias	9	Describe any efforts to address potential sources of bias	12	It is s a cross-sectional character, coupled with the self-reported health status does not allow to exclude the presence of active infections in some

				individuals which may increase the level of ROS. The second limitation is inherent to the nature of the d-ROMs measurement, which only provides a proxy of the oxidative status, being a measure of the total peroxy-derivatives present in the plasma and not a direct measure of ROS.
Study size	10	Explain how the study size was arrived at	6	Only people who fulfilled the criteria selection were selected within the sampling periods.

Continued on next page

Quantitative variables	11	Explain how quantitative variables were handled in the analyses. If applicable, describe which groupings were chosen and why	6	Measurement of the d-ROMs<br>Measurement of the PAT<br>Statistical Analysis
Statistical methods	12	(*a*) Describe all statistical methods, including those used to control for confounding	6	Statistical Analysis
		(*b*) Describe any methods used to examine subgroups and interactions		
		(*c*) Explain how missing data were addressed		
		(*d*) *Cohort study*—If applicable, explain how loss to follow-up was addressed<br>*Case-control study*—If applicable, explain how matching of cases and controls was addressed<br>*Cross-sectional study*—If applicable, describe analytical methods taking account of sampling strategy	6	Measurement of the d-ROMs<br>Measurement of the PAT<br>Statistical Analysis
		(*e*) Describe any sensitivity analyses		
**Results**				
Participants	13*	(a) Report numbers of individuals at each stage of study—eg numbers potentially eligible, examined for eligibility, confirmed eligible, included in the study, completing follow-up, and analysed	6	Demographic analysis of the sampled populations.
		(b) Give reasons for non-participation at each stage		
		(c) Consider use of a flow diagram		
Descriptive data	14*	(a) Give characteristics of study participants (eg demographic, clinical, social) and information on exposures and potential confounders	6	Demographic analysis of the sampled populations.
		(b) Indicate number of participants with missing data for each variable of interest		
		(c) *Cohort study*—Summarise follow-up time (eg, average and total amount)		
Outcome data	15*	*Cohort study*—Report numbers of outcome events or summary measures over time		
		Case-control study—Report numbers in each exposure category, or summary measures of exposure		
		Cross-sectional study—Report numbers of outcome events or summary measures	*7*	d-ROMs test<br>PAT test
Main results	16	(*a*) Give unadjusted estimates and, if applicable, confounder-adjusted estimates and their precision (eg, 95% confidence interval). Make clear which confounders were adjusted for and why they were included	7-8	d-ROMs test<br>PAT test<br>Gender differences
		(*b*) Report category boundaries when continuous variables were categorized		
		(*c*) If relevant, consider translating estimates of relative risk into absolute risk for a meaningful time period		

Continued on next page

Other analyses	17	Report other analyses done—eg analyses of subgroups and interactions, and sensitivity analyses	8	Post-hoc analysis
**Discussion**				
Key results	18	Summarise key results with reference to study objectives	11-12	The first clear indication we obtained is that the oxidative status, as measured by the d-ROMs parameter, is significantly higher than that observed in analogous population-wide studies in Europe or in East Asia.<br>When the antioxidant potential, as measured by the PAT test, is evaluated, it is centered around a value of 2853.6 UCOR, with a standard deviation of 635.7. This is still within the range of normality accepted for Caucasian populations but at the upper level. This can be tentatively interpreted as an adaptation to a more oxidant environment: people produce larger amounts of ROS but have a higher ability to quench them.
Limitations	19	Discuss limitations of the study, taking into account sources of potential bias or imprecision. Discuss both direction and magnitude of any potential bias	12	This study has some limitations. It is s a cross-sectional character, coupled with the self-reported health status does not allow to exclude the presence of active infections in some individuals which may increase the level of ROS. The second limitation is inherent to the nature of the d-

				ROMs measurement, which only provides a proxy of the oxidative status, being a measure of the total peroxy-derivatives present in the plasma and not a direct measure of ROS.
Interpretation	20	Give a cautious overall interpretation of results considering objectives, limitations, multiplicity of analyses, results from similar studies, and other relevant evidence	12	The large amount of the sampled population, and the robust statistics we obtained, make us confident that the observed measurements can effectively be taken as a baseline value for further screening purposes,
Generalisability	21	Discuss the generalisability (external validity) of the study results	12	The results can be used either in the context of the COVID-19 pandemic or in other conditions, such as malnutrition or other noncommunicable chronic diseases, with the aim of productive population stratification and correct use of available medical resources.
**Other information**				
Funding	22	Give the source of funding and the role of the funders for the present study and, if applicable, for the original study on which the present article is based	13	National Council for Science and Technology of Rwanda

*Give information separately for cases and controls in case-control studies and, if applicable, for exposed and unexposed groups in cohort and cross-sectional studies.

Note: An Explanation and Elaboration article discusses each checklist item and gives methodological background and published examples of transparent reporting. The STROBE checklist is best used in conjunction with this article (freely available on the Web sites of PLoS Medicine at http://www.plosmedicine.org/, Annals of Internal Medicine at http://www.annals.org/, and Epidemiology at http://www.epidem.com/). Information on the STROBE Initiative is available at www.strobe-statement.org.
