# Supplementary material for: Evaluation of oxidative stress markers in Rwanda during the SARS-CoV-2 pandemic: A cross-sectional study
Source: PLOS Glob Public Health. 2023 Oct 25;3(10):e0002487. doi: 10.1371/journal.pgph.0002487 (PMC10599508; doi:10.1371/journal.pgph.0002487)
Supplement: S1 File — (PDF) [file pgph.0002487.s004.pdf]

REPUBLIC OF RWANDA/REPUBLIQUE DU RWANDA

NATIONAL ETHICS COMMITTEE / COMITE NATIONAL D'ETHIQUE

Telephone: (250) 2 55 10 78 84
E-mail: info@rnecrwanda.org
Web site: www.rnecrwanda.org

Ministry of Health
P.O. Box. 84
Kigali, Rwanda.

FWA Assurance No. 00001973
IRB 00001497 of IORG0001100

March 23rd ,2021.
No.91/RNEC/2021

Project Principal Investigator: Dr. Thierry HABYARIMANA
INSTITUT D'ENSEIGNEMENT SUPÉRIEUR DE RUHENGERI (INES)

Your research project: **"Predicting the risk of SARS-Cov-2 infection and co-morbidity and Reducing Socioeconomic Impacts: Identification of high risk population"** has been evaluated by the Rwanda National Ethics committee.

Name	Institute	Involved in the decision		
		Yes	No (Reason)	
			Absent	Withdrawn from the proceeding
Dr. Jean-Baptiste MAZARATI	Chairperson of the RNEC		X	
Prof. Jean Paul RWABIHAMA	University of Rwanda		X	
Prof.Laetitia NYIRAZINYOYE	University of Rwanda	X		
Ass .Prof. Egide KAYITARE	University of Rwanda	X		
Mr. Spencer BUGINGO	Lawyer		X	
Ass. Prof. David K. TUMUSIIME	University of Rwanda	X		
Ass. Prof. Lisine TUYISENGE	Kigali Teaching Hospital	X		
Dr. Darius GISHOMA	University of Rwanda	X		
Sr.Epiphanie MUKABARANGA	Rwamagana Nursing and Midwife school		X	
Dr. Vedaste NDAHINDWA	University of Rwanda	X		
Prof. Claude MUVUNYI	Biomedical Services (BIOS)	X		

After review of the protocol and consent forms, during the RNEC meeting of 19th February 2021 where quorum was met, and revisions made on the advice of the RNEC submitted on 22nd March 2021, **we hereby provide approval for the above-mentioned protocol**.

Please note that approval of the protocol and consent form both English and Kinyarwanda version is valid for **12 months**.

You are responsible for fulfilling the following requirements:

1. Changes, amendments, and addenda to the protocol or consent form must be submitted to the committee for review and approval, prior to activation of the changes.
2. Only approved consent forms are to be used in the enrollment of participants
3. All consent forms signed by subjects should be retained on file. The RNEC may conduct audits of all study records, and consent documentation may be part of such audits.
4. A continuing review application must be submitted to the RNEC in a timely fashion and before expiry of this approval.
5. Failure to submit a continuing review application will result in termination of the study.
6. Notify the Rwanda National Ethics committee once the study is completed.

Sincerely,

RWANDA NATIONAL ETHICS COMMITTEE R.N.E.C P.O BOX [illegible] KIGALI

Dr. Jean- Baptiste MAZARATI
Chairperson, Rwanda National Ethics Committee.

Date of Approval: March 23,2021
Expiration date: March 22,2022

C.C.
- Hon. Minister of Health.
- The Permanent Secretary, Ministry of Health.
