# Supplementary material for: Evaluation of oxidative stress markers in Rwanda during the SARS-CoV-2 pandemic: A cross-sectional study
Source: PLOS Glob Public Health. 2023 Oct 25;3(10):e0002487. doi: 10.1371/journal.pgph.0002487 (PMC10599508; doi:10.1371/journal.pgph.0002487)
Supplement: S2 File — (PDF) [file pgph.0002487.s005.pdf]

REPUBLIC OF RWANDA

NORTHERN PROVINCE
P.O. BOX: 233 MUSANZE
E-mail northern@northernprovince.gov.rw

Musanze on, 19th February 2021

Ref: 308/07.04/AAG/2021

To: The Vice Chancellor
of INES-Ruhengeri

RE: Authorization to conduct a Research Project

Dear Sir,

Reference is made to your letter dated 18th February 2021, asked the Autorisation to conduct a Reseach Project on Covid-19; Following the award of Research Grant by INES-Ruhengeri on the project "***Predicting the risk of SARS-Cov-2 infection and co-morbidity and Reducing Socioeconomic Impacts: Identification of high risk population***", under the theme: "*Special Collaborative Research Grants to Address COVID-19 Pandemic*", funded by National Council for Science and Technology,

I hereby inform you that you are authorized to conduct and collect the required data/samples for the project in Musanze, Gakenke and Burera Districts respecting the research standards. Competitive authorities in respective Districts will facilitate and smoothen the implementation of the project.

Wish you all the success.

Jean Marie Vianney GATABAZI
Governor of Northern Province

CC:
The Executive Secretary of Northern Province/**MUSANZE**
The Mayor of Musanze
The Mayor of Gakenke
The Mayor of Burera
